# Reforming Nitrate Metabolism for Enhancing L-Arginine Production in *Corynebacterium crenatum* Under Oxygen Limitation

**DOI:** 10.3389/fmicb.2022.834311

**Published:** 2022-03-09

**Authors:** Mingzhu Huang, Lingfeng Zhu, Lin Feng, Li Zhan, Yue Zhao, Xuelan Chen

**Affiliations:** ^1^Department of Life Science, Jiangxi Normal University, Nanchang, China; ^2^National R&D Center for Freshwater Fish Processing, Nanchang, China; ^3^Key Laboratory of Functional Small Organic Molecule of Ministry of Education, Jiangxi Normal University, Nanchang, China

**Keywords:** L-arginine, *Corynebacterium crenatum*, nitrate metabolism, anaerobic production, nitrate respiration

## Abstract

Various amino acids are widely manufactured using engineered bacteria. It is crucial to keep the dissolved oxygen at a certain level during fermentation, but accompanied by many disadvantages, such as high energy consumption, reactive oxygen species, and risk of phage infections. Thus, anaerobic production of amino acids is worth attempting. Nitrate respiration systems use nitrate as an electron acceptor under anoxic conditions, which is different from the metabolism of fermentation and can produce energy efficiently. Herein, we engineered *Corynebacterium crenatum* to enhance L-arginine production under anaerobic conditions through strengthening nitrate respiration and reforming nitrogen flux. The construction of mutant strain produced up to 3.84 g/L L-arginine under oxygen limitation with nitrate, and this value was 131.33% higher than that produced by the control strain under limited concentrations of oxygen without nitrate. Results could provide fundamental information for improving L-arginine production by metabolic engineering of *C. crenatum* under oxygen limitation.

## Highlights

– To produce amino acids under oxygen limitation.
– To fermentation using nitrate as a terminal electron.

## Introduction

Various amino acids, including arginine, histidine, tryptophan, and valine, are widely manufactured using regulatory mutants of *Corynebacterium glutamicum* (Rehm and Burkovski, 2011). For amino acid production, the dissolved oxygen (DO) should be kept at a certain level during fermentation (Yasuda et al., 2007; Okino et al., 2008). To this end, the general strategy is to change the aeration and stirring speed of bioreactor during fermentation. However, high oxygen supply, which is obtained by injecting pure oxygen or increasing the agitation speed, requires high energy consumption during the fermentation and thus increases costs. Furthermore, production rates of reactive oxygen species (ROS) are proportional to those of DO (Korshunov and Imlay, 2006), and cells are exposed to hyperbaric oxygen that often surpasses the air saturation during aerobic industrial fermentations, which is detrimental for amino acid biosynthesis (Imlay, 2002). Notably, the most common source of bacteriophages is overpressurized air used to control fermentation, which may lead to destruction of fermentation medium and subsequent contamination of the facility and thus heavy financial loses (Los, 2012). If we can breed strains that produce amino acids efficiently even under anaerobic conditions, then a significant advance would likely arise in the fermentation industry.

Bacteria that grow in environments where oxygen is absent, such as obligate and facultative anaerobes, possess various anaerobic metabolism modes, which are divided into two: “anaerobic respiration” and “fermentation” (Takeno et al., 2007). In a typical “fermentation,” the amount of ATP depends on the level of substrate phosphorylation. The production rate of ATP is low, cell growth is decreased correspondingly, and most of carbon flux is directed to undesirable organic acids without any electron acceptors (Toya et al., 2012). Thus, achieving efficient production of amino acid in parallel to fermentative metabolism is difficult. However, many microorganisms can realize anaerobic respiration by utilizing various compounds other than oxygen as terminal electron acceptors to provide sufficient energy for serving the end products (Reiner et al., 1999). For example, many bacteria possess nitrate respiration systems that use nitrate as an electron acceptor under anoxic conditions (Kraft et al., 2011). *C. glutamicum* can grow under oxygen limitation using nitrate as a terminal electron acceptor as well (Nishimura et al., 2007). *C. glutamicum* utilizes nitrate and excretes nitrite as the main final product of nitrate reduction during anaerobic respiration. This anaerobic respiration is attributed to a *narKGHJI* operon, which is induced under anaerobic conditions in the presence of nitrate (Nishimura et al., 2007); ArnR is a repressor of the *narKGHJI* gene clusters under anaerobic conditions (Nishimura et al., 2008). Takeno et al. (2007) confirmed the possibility of any amino acid production by nitrate respiration in *C. glutamicum* owing to that the TCA cycle is operative during anaerobic nitrate respiration.

**FIGURE 1:**
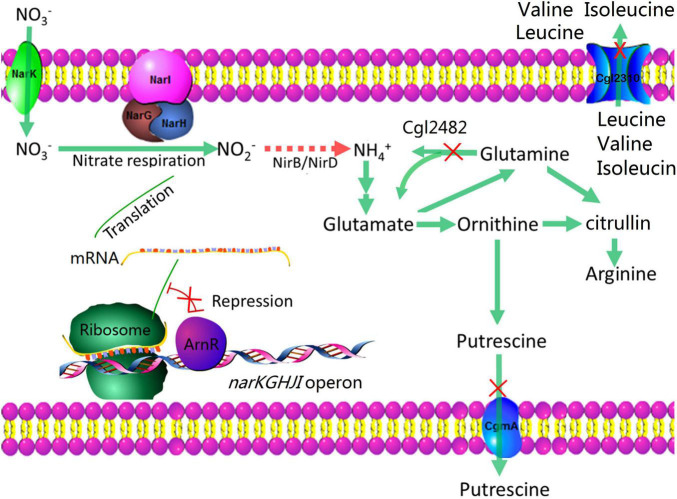
Metabolic strategies for enhancing L-arginine production. The red crosses indicate that the genes are disrupted. The arrows indicate the pathways of nitrogen fluxes; the red arrows indicate heterologous overexpression of nitrite reductase for reducing nitrite to ammonia.

*Corynebacterium crenatum*, which is a close relative of *C. glutamicum*, has a long history as a producer of various amino acids (Su et al., 2014). In this study, we engineered M00 (*C. crenatum* AS 1.542 mutation strain with auxotrophic for biotin, and producing L-arginine) to enhance L-arginine production under anaerobic conditions through nitrate addition for strengthening nitrate respiration and reforming nitrogen flux. We first studied the effects of nitrate addition on L-arginine production and found that the addition of nitrate was beneficial for L-arginine production under anaerobic conditions. Then, we disrupted *arnR* to increase the expression of the *narKGHJI* operon. We also reduced nitrite to ammonia through heterologous expression of nitrite reductase genes, which resulted in higher growth rate under limited concentrations of oxygen. To introduce the nitrogen flux into L-arginine biosynthesis, *cgmA*, *cgl2310*, and *cgl2482* were disrupted (Figure 1). Finally, the constructed mutant strain (M00 Δ*arnR*Δ*cgmA* Δ*cgl2310*Δ*cgl2482*: *nirBD*) produced up to 3.84 g/L L-arginine under limited concentrations of oxygen with nitrate, and this value was 131.33% higher than that produced by the control strain under limited concentrations of oxygen without nitrate.

## Materials and Methods

### Construction of Plasmids and Strains

A list of all strains and plasmids used in this study can be found in Table 1. For amplification of specific genes, the genomic DNA of *C. crenatum* and *Escherichia coli* were isolated and then used as the template. The suicide vector pK18mobsacB was used for markerless gene insertion and deletion as described previously (Shäfer et al., 1994). *E. coli* DH5α was used for recombinant plasmid construction and amplification. For gene deletion, the upstream and downstream fragments of *arnR*, *cgmA*, *cgl2482*, and *cgl2310* were amplified using polymerase chain reaction (PCR). Subsequently, the upstream and downstream fragments were overlapped using overlapping PCR and inserted into pK18*mobsacB* by NovoRec^®^Plus PRC kit (Novoprotein, China). The coding sequence of *nirBD* was amplified by PCR using genomic DNA of *E. coli*. The coding sequence of *nirBD* was inserted into pXMJ19. All recombinant plasmids were transformed into *C. crenatum* and *C. crenatum*-derived strains by electroporation. The mutants were checked using colony PCR.

**TABLE 1:** Strains and plasmids in this study.

Strains/plasmids	Characteristics	Resources
Cplamids		
pK18*mobsacB*	Mobilizable vector, allows for selection of double crossover in *C. crenatum*, Km^R^, *sacB*	Biovector
pK18*mobsacB*-Δ*arnR*	A derivative of pK18*mobsacB*, harboring Δ *arnR* fragments	This work
pK18*mobsacB*-Δ*cgmA*	A derivative of pK18*mobsacB*, harboring Δ *cgmA* fragments	This work
pK18*mobsacB*-Δ *cgl2310*	A derivative of pK18*mobsacB*, harboring Δ *cgl2310* fragments	This work
pK18*mobsacB*-Δ*cgl2482*	A derivative of pK18*mobsacB*, harboring Δ *cgl2482* fragments	This work
pXMJ19	Shuttle vector for overexpression, Chl^R^	Biovector
pXMJ19- *nirBD*	A derivative of pXMJ19, harboring *nirBD* gene fragments from *E. coli*	This work
strains		
*E. coli* DH5α	Clone host strain	Invitrogen
M00	*C. crenatum* AS 1.542 mutation strain with auxotrophic for biotin, and producing L- arginine	Lab Stock (Chen et al., 2015)
M01	M00 with a deletion of the *arnR* gene	This work
M02	M00 harboring pXMJ19- *nirBD*	This work
M03	M01 harboring pXMJ19- *nirBD*	This work
M04	M03 with a deletion of the *cgmA* gene	This work
M05	M03 with a deletion of the *cgl2482* gene	This work
M06	M03 with a deletion of the *cgl2310* gene	This work
M07	M06 with a deletion of the *cgmA* gene	This work
M08	M06 with a deletion of the *cgl2482* gene	This work

### Medium and Cultivation Conditions

Luria–Bertani (LB) medium (NaCl 10 g L^–1^, yeast extract 5 g L^–1^, tryptone 10 g L^–1^, pH 7.0) was used to propagate *E. coli* and *C. crenatum*, and 10 μg ml^–1^ chloromycetin and 50 μg ml^–1^ kanamycin were supplemented as necessary. Competent *C. crenatum* cells for electroporation were prepared by cultivating *C. crenatum* strains in competent medium (LB medium supplemented with 30 g L^–1^ glycine) to OD600 of 1.5 and washing cells three times with 15% (v/v) glycerol. Sucrose medium (LB medium supplemented with 25 g L^–1^ sucrose) was used to remove the suicide plasmid pK18*mobsacB* containing the sucrose lethal gene *sacB* (Zhang et al., 2018). Growth analyses under oxygen limited conditions were performed using mineral salt medium (BT medium) containing (per liter) 7 g (NH_4_)_2_SO_4_, 2 g of urea, 0.5 g K_2_HPO_4_, 0.5 g KH_2_PO_4_, 0.5 g MgSO_4_⋅7H_2_O, 4.2 mg MnSO_4_⋅H_2_O, 6 mg FeSO_4_⋅7H_2_O, 0.2 mg of thiamine, and 0.2 mg of biotin. The precultivations of *C. crenatum* strains were cultivated at 30°C in LBG medium (LB medium supplemented with 5 g L^–1^ glucose). The seed medium (per liter) of *C. crenatum* consisted of 30 g of glucose, 1.5 g of urea, 0.5 g of MgSO_4_⋅7H_2_O, 1 g of KH_2_PO_4_, and pH 7.0 (Chen et al., 2015). The fermentation medium (per liter) was composed of 100 g of glucose, 1.5 g of urea, 0.5 g of MgSO_4_⋅7H_2_O, 40g (NH_4_)_2_SO_4_ 1 g of KH_2_PO_4_, 0.05 g of MnSO_4_⋅2H_2_O, 0.02 g of FeSO_4_⋅7H_2_O, 30 g of CaCO_3_, 8 × 10^–5^ g of biotin, 5 × 10^–4^ g of thiamine, pH 7.0, and added with an appropriate concentration nitrate as necessary. For oxygen limitation cultivation, *C. crenatum* strains were grown in a 250 ml flask covered with rubber stopper. The microaerobic conditions during fermentation were confirmed by the oxygen indicator resazurin (Nishimura et al., 2007). To induce heterologous gene expression, isopropyl-β-D-thiogalactopyranoside (IPTG) was added.

### Cell Growth Analyses Under Oxygen Limited Conditions

The strains were cultured in LB medium for approximately 18 h under anaerobic conditions. Subsequently, the strains were transferred into 50 ml of fresh BT medium with 100 mM potassium nitrate in a 250 ml flask covered with rubber stopper at an initial OD at 562 nm of 0.06; then, the bottle was flushed with sterile nitrogen gas for 30 min (Chen et al., 2015). Sampling was conducted aseptically under constant flushing with nitrogen.

### Fermentation in Shake Flasks

The strains were activated on LB agar plate for 24 h. Subsequently, the activated strains were cultured in 10 mL seed medium at 30°C for 18 h with agitation at 200 rpm. Approximately 2 mL of each seed medium was transferred into 25 mL fermentation medium in 250 ml flask with rubber stopper, and then, the bottle was flushed with sterile nitrogen gas for 30 min (Laura et al., 2014). All fermentation cultures were incubated at 30°C and 200 rpm, and samples were collected every 12 h for measurement of cell density, L-arginine, organic acid, and residual glucose under constant flushing with nitrogen.

### Optimal Nitrate and Isopropyl-β-D-Thiogalactopyranoside Concentrations for L-Arginine Fermentation Under Oxygen Limitation

The strains were activated on LB agar plate for 24 h. Subsequently, the activated strains were cultured in 10 mL seed medium at 30°C for 18 h with agitation at 200 rpm. Approximately 0.1 mL of each seed medium was transferred into 2 mL fermentation medium with different concentrations of IPTG and nitrate in test tubes with rubber stopper, and then, the bottle was flushed with sterile nitrogen gas for 30 min (Laura et al., 2014). All fermentation cultures were incubated at 30°C and 200 rpm for 36 h.

### RT-PCR Analysis

M00 and M00 Δ*arnR* (M01) cells were cultured to mid-log phase anaerobically with nitrate. The cells were harvested for total RNA extracted by Bacteria Total RNA Isolation Kit (Sangon Biotech, Shanghai, China) in accordance with the instructions of the manufacturer. Reverse transcription was conducted by the PrimeScript™ RT Reagent Kit with gDNA Eraser (TaKaRa, Beijing, China). Real-time PCR (RT-PCR) analyses were conducted as described previously (Liao et al., 2015).

### Analytical Methods

The concentration of L-arginine was measured using a Sykam S-433D amino acid analyzer (Sykam Co., Ltd., Germany) (Zhang et al., 2018). Glucose concentrations were determined enzymatically by a bioanalyzer (SBA-40E, Shandong, China). Cell growth was determined by measuring the OD562 (1 OD562 = 0.375 g L^–1^ dry cell weight) with a spectrophotometer (BG-XM496, Beijing, China). Nitrite concentrations were determined using Nitrite Content Assay Kit (Solarbio, Beijing, China). NADPH concentrations were determined using NADPH Content Assay Kit (Solarbio, Beijing, China). ATP concentrations were determined using ATP Content Assay Kit (Solarbio, Beijing, China). NADH concentrations were determined using NADH Content Assay Kit (Beyotime, Shanghai, China). All experiments were performed in triplicates, and data were represented as the mean ± standard deviation.

## Results

### Influence of Nitrate Addition on L-Arginine Fermentation Under Oxygen Limitation

**FIGURE 2:**
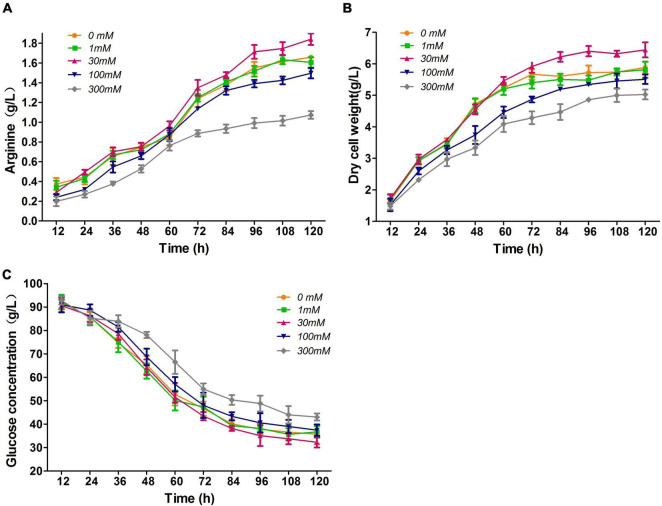
Effects of nitrate addition on L-arginine fermentation under oxygen limitation. **(A)** L-arginine production. **(B)** Dry cell weight. **(C)** Glucose concentration. Samples were collected per 12 h for the determination of fermentation parameters. Data represent average values and results of standard deviations present in three individual experiments.

First, we investigated the effect of nitrate addition concentrations of 0, 1, 30, 100, and 300 mM on L-arginine fermentation of M00 under oxygen limitation. At the nitrate concentrations of 100 and 300 mM, the final L-arginine production rates were reduced by 9.6 and 35.5%, respectively; nevertheless, cultures with 30 mM nitrate fermented 10.8% higher concentrations of L-arginine than that without nitrate (Figure 2A). The cultures grown at 30 mM nitrate formed significant higher cell density (Figure 2B). The glucose consumptions only showed slight differences at nitrate concentrations of 0–30 mM (Figure 2C), and the glucose consumptions of cultures with more than 100 mM were reduced compared with that in the control.

### Engineering of Nitrate Metabolism

**FIGURE 3:**
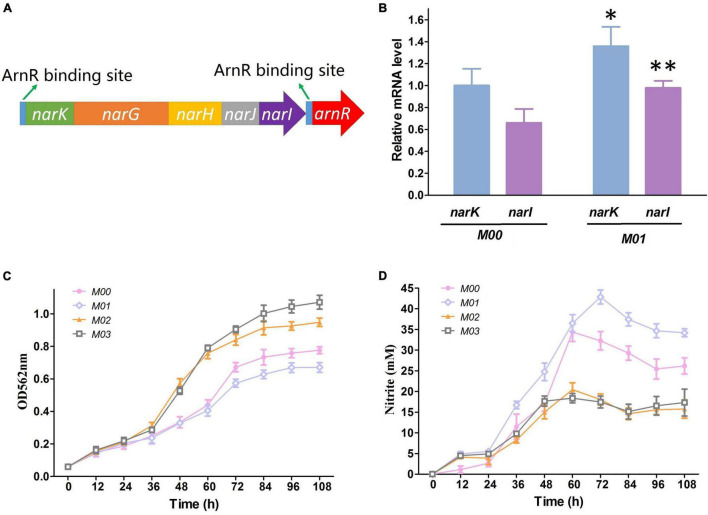
Effect of *arnR* disruption and *nirBD* overexpression on nitrate respiration. **(A)** Gene arrangements of the *narKGHJI* operon and the *arnR* gene. **(B)**. Relative transcription level of *narK* and narI in M00 and M01. **(C)** Growth in glucose minimal medium under anaerobic conditions with presence of 100 mM nitrate. **(D)** Nitrite concentration in glucose minimal medium under anaerobic conditions with presence of 100 mM nitrate. Data represent average values and results of standard deviations present in three individual experiments. The differences among groups were determined by ANOVA. Differences were considered statistically significant at *p* < 0.1. “^**^” indicates *p* < 0.05; “*” indicates 0.05 < *p* < 0.1.

Nitrate respiration is an important physiological process that allows bacteria to generate sufficient energy for enabling anaerobic growth. *C. crenatum* possesses *narKGHJI* operon, and the repressor gene *arnR* is located immediately downstream of the operon (Figure 3A). ArnR regulates the expression of the *narKGHJI* operon through binding the upstream of the transcriptional start sites. Thus, the *arnR* gene was deleted in this study for further enhancement in the *narKGHJI* operon expression. The mRNA levels of the *narK* and *narI* genes were used as representative expression level markers of the *narKGHJI* operon. Transcriptional levels of *narK* and *narI* in M00Δ*arnR* (M01) were apparently higher than those in M00 under anaerobic growth with nitrate (Figure 3B).

The *narKGHJI* operon could only reduce nitrate to harmful nitrite but not up to ammonia, and ammonium is a preferred nitrogen source for many microorganisms (Rehm and Burkovski, 2011). For this reason, the *nirBD*-encoded nitrite reductase (Nir) was introduced from *E. coli*. We performed growth experiments using M00, M01, M00:*nirBD* (M02), and M00Δ*arnR*:*nirBD* (M03) in BT minimum medium with the presence of a relatively high nitrate concentration (100 mM) under anaerobic conditions to probe the influence of gene modification on anaerobic growth rate and nitrate metabolism. OD at 562 nm and nitrite concentrations in the culture medium were measured. The growth of M01 showed the weakest growth rate under anaerobic conditions with nitrate (Figure 3C), which indicated that *arnR* disruption contributed to the *narKGHJI* operon overexpression (Figure 3B) and then formed higher concentrations of nitrite (Figure 3D) that resulted in growth inhibition. Nitrate respiration allowed M02 and M03 to generate sufficient energy for enabling anaerobic growth, and Nir detoxified nitrite that accumulated from nitrate respiration (Figure 3D). Therefore, the growth rates of M02 and M03 were significantly increased compared with that of M00 (Figure 3C). The growth rate of M03 was higher than that of M02 (Figure 3C). The reason may be that *arnR* disruption improved the flux of nitrate respiration to generate sufficient energy for growth and the overexpression nitrite reductase was sufficient to eliminate excess nitrite.

**FIGURE 4:**
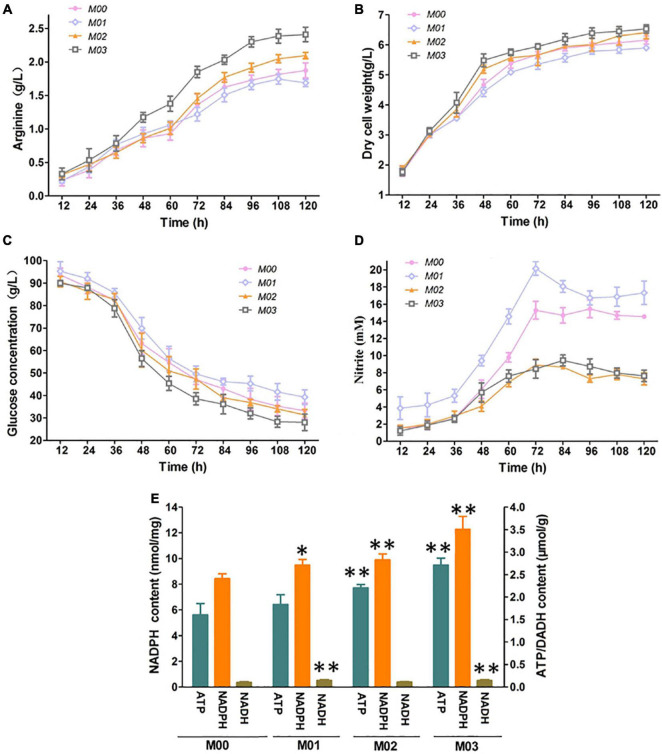
Effect of *arnR* disruption and *nirBD* overexpression L-arginine fermentation under oxygen limitation with 30 mM nitrate. **(A)** L-arginine production. **(B)** Dry cell weight. **(C)** Glucose concentration. **(D)** Nitrite concentration. **(E)** NADPH, NADH and ATP of the samples at log phase. Samples were collected per 12 h for the determination of fermentation parameters. Data represent average values and results of standard deviations present in three individual experiments. The differences among groups were determined by ANOVA. Differences were considered statistically significant at *p* < 0.1. “^**^” indicates *p* < 0.05; “*” indicates 0.05 < *p* < 0.1.

We further examined the effect of *arnR* disruption and *nirBD* overexpression on L-Arginine production under oxygen limitation with 30 mM nitrate. As shown in Figure 4, M01 exhibited the lowest arginine titer, dry cell weight, and glucose consumption compared with those of others. However, M01 formed the highest concentrations of nitrite, which implied that high concentrations of nitrite created by the high-intensity nitrate respiration seriously impaired fermentation for L-arginine due to *arnR* disruption. M02 and M03 exhibited lower concentrations of nitrite than M00 as a result of *nirBD* overexpression. M02 and M03 exhibited higher arginine titer, and the L-arginine production of M03 was 28.8% higher than that of M00. In addition, the dry cell weight and glucose consumption of M03 were higher than those of M00. NADPH, NADH and ATP are involved in many cellular physiological activities and metabolic pathways, and they have a vital role in production of nearly all of the metabolites using industrial strains (Zhou et al., 2009). Increasing NADPH, NADH and ATP supply is a powerful tool to achieve higher yield on L-arginine (Man et al., 2016). We measured the NADPH, NADH and ATP of the samples at log phase (36 h). As shown in Figure 4E, M01 obtained higher NADPH and NADH content than M00; two strains harboring *nirBD* gene (M02 and M03) showed significantly higher content of NADPH and ATP than M00. The strain with *nirBD* gene overexpression and *arnR* disruption (M03) showed significantly higher content of NADPH, NADP and ATP than M00. M03 is the best for efficient L-arginine production during anaerobic growth. Therefore, M03 was used for further experiments.

### Optimal Nitrate and Isopropyl-β-D-Thiogalactopyranoside Concentrations of M03 for L-Arginine Fermentation Under Oxygen Limitation

**FIGURE 5:**
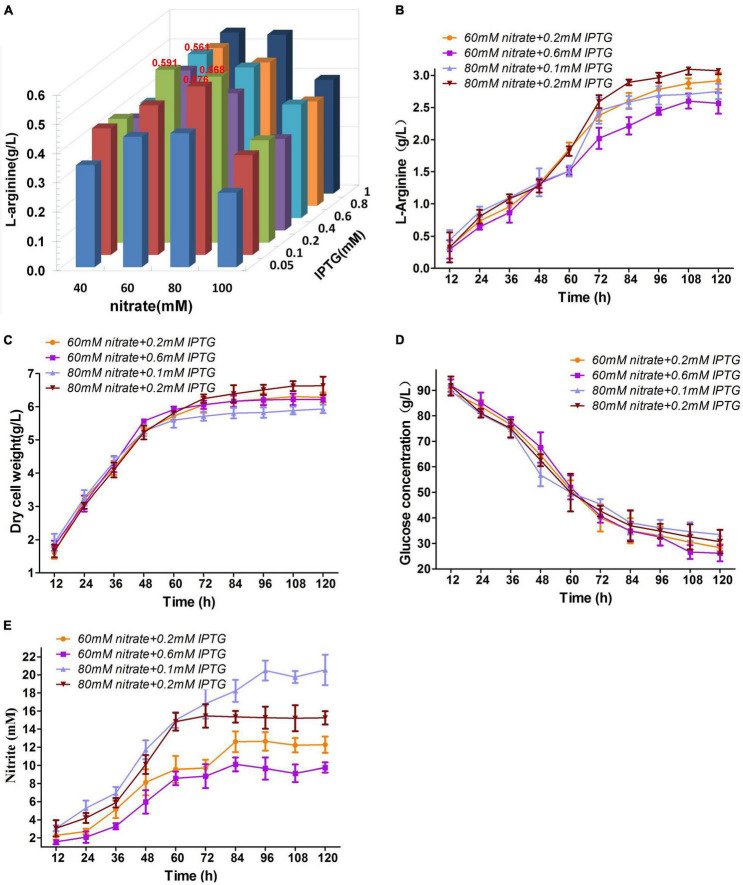
Optimal nitrate and IPTG concentrations. **(A)** Orthogonal experiment of nitrate and IPTG concentrations for M03. **(B)** L-arginine production. **(C)** Dry cell weight. **(D)** Glucose concentration. **(E)** Nitrite concentration. Samples were collected per 12 h for the determination of fermentation parameters. Data represent average values and results of standard deviations present in three individual experiments.

M03 maintained low nitrite levels during flask cultivation under oxygen limitation with 30 mM nitrate (Figure 4D), and more IPTG induced more *nirBD* gene expression to reduce nitrite to ammonium. Therefore, the optimal nitrate and IPTG concentrations could further strengthen nitrate respiration and could thus exhibit higher growth rate and L-arginine production. To probe the influence of nitrate and IPTG concentrations on L−arginine production under anaerobic conditions, M03 was cultured in test tube with 2 mL fermentation medium and different concentrations of IPTG (from 0.05 to 1 mM) and nitrate (from 40 to 100 mM) under oxygen limitation for 36 h. The presence of 60 mM nitrate + 0.2 mM IPTG represented the highest L-arginine yield; the presence of 60 mM nitrate + 0.6 mM IPTG, 80 mM nitrate + 0.1 mM IPTG, and 80 mM nitrate + 0.2 mM IPTG produced relatively high L-arginine yield as well (Figure 5A). Then, the shake flask fermentation with the four combinations mentioned above of nitrate and IPTG were examined (Figure 5). No significant difference was found in glucose consumption of the four combinations (Figure 5D). The L-arginine production was the highest with presence of 80 mM nitrate + 0.2 mM IPTG, which produced 3.07 g/L L-arginine (Figure 5B); the presence of 80 mM nitrate + 0.2 mM IPTG also performed the highest dry cell weight (Figure 5C); 0.1 mM IPTG may induce lower *nirBD* gene expression and thus high concentration of nitrite (Figure 5E), which was harmful to cell growth and L-arginine production; for this reason, the presence of 80 mM nitrate + 0.1 mM IPTG performed the lowest dry cell weight and relatively lower L-arginine production (Figures 5B,C). The two combinations with 60 mM nitrate performed the lower nitrite concentration (Figure 5E) but may be insufficient to nitrate respiration intensity for high L-arginine production. Therefore, we used 80 mM nitrate + 0.2 mM IPTG in further cultivation studies below.

### Nitrogen Flux Adjustment

**FIGURE 6:**
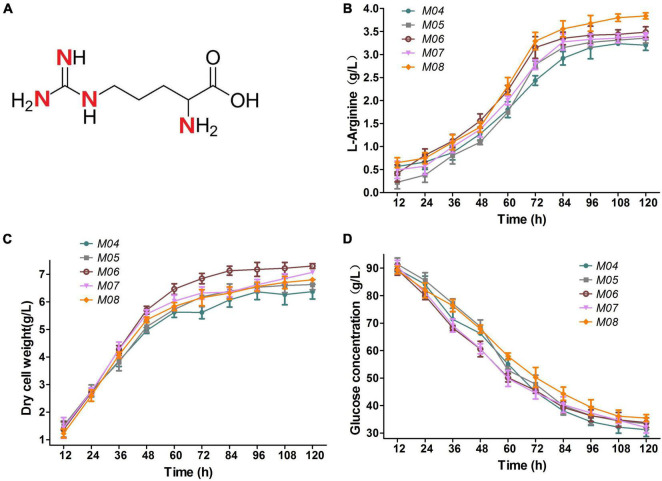
**(A)** L-arginine molecule. **(B)** L-arginine production. **(C)** Dry cell weight. **(D)** Glucose concentration. Samples were collected per 12 h for the determination of fermentation parameters. Data represent average values and results of standard deviations present in three individual experiments.

L-arginine molecule possesses four nitrogen atoms and has a nitrogen content up to 32.1% (Figure 6A). Improvement in the nitrogen assimilation is a popular and important way to enhance L-arginine (Guo et al., 2017). A putrescine transporter encoded by *cgmA* and a branched-chain amino acid permease encoded by *cgl2310* were deleted to avoid intracellular nitrogen loss for further increasing L-arginine production by nitrogen flux adjustment illustrated in Figure 1. The two constructed strains were named M04 and M06, respectively. Glutaminase encoded by *cgl2482* catalyzes the formation of glutamate and ammonium from glutamine. M05 was constructed by *cgl2482* deletion to push nitrogen flux toward L-arginine. During shake flask anaerobic fermentation for 120 h, M06 performed the highest dry cell weight due to *cgl2310* deletion to avoid intracellular nitrogen loss (Figure 6C), and the glucose consumption of M05 and M06 improved slightly compared with that of M04 (Figure 6D). Notably, the glucose consumption of M04, M05, and M06 were reduced compared with that of M03, but the L-arginine production rates of M04, M05, and M06 increased by 4.2, 10.7, and 13.4% compared with that produced by the strain M03, respectively (Figure 6), which indicated that the deletion of *cgmA*, *cgl2482*, and *cgl2310* improved the utilization of the glucose. Among which, the *cgl2310* deletion stain (M06) resulted in 3.48 g/L L-arginine, which was the highest L-arginine production rate. Thus, M06 with *cgmA* or *cgl2482* deletion (named M07 and M08, respectively) were constructed to test the combinational effect. However, M07 produced 3.40 g/L L-arginine, which indicated a slight reduction in L-arginine production compared with that observed in M06 (Figure 6B). M08 produced 3.84 g/L L-arginine, which was 10.3% higher than that produced by M06 (Figure 6B); M08 also performed the highest L-arginine yield per biomass and glucose.

## Discussion

In this study, we engineered *C. crenatum* to enhance L-arginine production under anaerobic conditions. We have demonstrated in this study that the addition of nitrate was beneficial for L-arginine production under anaerobic conditions. The *arnR* was disrupted to improve nitrate respiration. Heterologous expression of nitrite reductase genes resulted in higher growth rate under limited concentrations of oxygen. To introduce the nitrogen flux into L-arginine biosynthesis, *cgmA*, *cgl2310*, and *cgl2482* were disrupted, which resulted in L-arginine production further increased.

Under anaerobic conditions, the ATP production rate is low. ATP is formed by substrate level phosphorylation, and cell growth rate is therefore significantly decreased without oxygen (Toya et al., 2012). Notably, the respiratory nitrate reductases utilize nitrate as an electron acceptor that conducts the oxidation of NADH and concomitant ATP synthesis in anaerobic environments (Kraft et al., 2011). Moreover, the pentose phosphate (PP) pathway flux is increased under nitrate conditions, and the major role of the PP pathway is to supply NADPH for anabolism (Toya et al., 2012). In addition, intracellular NADPH, NADH and ATP levels are essential cofactors for L-arginine biosynthesis (Man et al., 2016). Thus, M00 grown at 30 mM nitrate had higher L-arginine production, glucose consumption rate, and dry cell weight. Similar to *C. glutamicum*, *C. crenatum* does not possess nitrite reductase; thus, nitrate can only be reduced to nitrite as the main end product during anaerobic growth (Nishimura et al., 2008). Nitrite have antimicrobial properties, which may be the reasons why M00 with excessive nitrate concentration (Figure 2B) exhibited lower L-arginine production, glucose consumption rate, and dry cell weight.

The further flux owing to *arnR* disruption increases the supply of NADPH, NADH and ATP. Moreover, Nir is necessary for nitrate/nitrite assimilation and has also been reported to either conduct nitrite detoxification or execute fermentative ammonification for anaerobic metabolism (Wang et al., 2019). Theoretically, *nirBD* gene introduced would be beneficial to anaerobic growth in the presence of nitrate, during which nitrite would be reduced to ammonium. Ammonification by Nir would be important for maximization of non-respiratory ATP production during anaerobic growth on nitrate. Intracellular NADPH, NADH and ATP levels are essential cofactors for L-arginine biosynthesis (Man et al., 2016). So, the strain with *nirBD* gene overexpression and *arnR* disruption (M03) also showed relatively high content of NADPH, NADH, ATP and L-arginine (Figure 4). Nitrate and nitrite reduction are important for anaerobic metabolism and nitrogen assimilation (Wang et al., 2019). On the contrary, *C. crenatum* lacking of any obvious counterpart to nitrite reductase could not reduce nitrite to ammonia; thus, the *nirBD* gene introduction opens up a new nitrogen assimilation pathway different from ammonium uptake systems for *C. crenatum*. In addition, the nitrate respiration improvement also increases nitrogen flux from nitrate. So, nitrogen flux adjustment is essential for L-arginine biosynthesis. Finally, *cgmA*, *cgl2482*, and *cgl2310* were deleted to improve L-arginine production by nitrogen flux adjustment. The construction of mutant strain produced up to 3.84 g/L L-arginine under limited concentrations of oxygen with nitrate, and this value was 131.33% higher than that produced by the control strain under limited concentrations of oxygen without nitrate. The dry cell weight and L-arginine production of anaerobic fermentation were lower than those of aerobic fermentation (Huang et al., 2020a). Intracellular NADPH, NADH and ATP levels were increased through nitrate reduction during anaerobic growth, but the NADPH, NADH and ATP levels were still lower than that in aerobic fermentation, which may be the reason for the decrease of arginine production and cells growth during anaerobic fermentation (Man et al., 2016; Huang et al., 2020b). Nevertheless, high oxygen supply lead to high energy consumption, production of reactive oxygen species, and bacteriophages contamination during fermentation. The research of produce amino acids efficiently under anaerobic conditions was significant for the fermentation industry. In the future, more efficient and low-cost anaerobic system should be used for industrial fermentation, eventually, anaerobic fermentation would reach the yield as aerobic. Our results could provide fundamental information for improving L-arginine production by metabolic engineering of *C. crenatum* under oxygen limitation.

## Data Availability Statement

The original contributions presented in the study are included in the article/supplementary material, further inquiries can be directed to the corresponding author/s.

## Author Contributions

MH and XC contributed to the research work and manuscript writing. MH contributed to the construction of plasmids and strains and bioinformatics analysis. YZ and LF contributed to the fermentation. LFZ and LZ contributed to the testing of RT-PCR analysis and analytical methods. All authors contributed to the article and approved the submitted version.

## Conflict of Interest

The authors declare that the research was conducted in the absence of any commercial or financial relationships that could be construed as a potential conflict of interest.

## Publisher’s Note

All claims expressed in this article are solely those of the authors and do not necessarily represent those of their affiliated organizations, or those of the publisher, the editors and the reviewers. Any product that may be evaluated in this article, or claim that may be made by its manufacturer, is not guaranteed or endorsed by the publisher.
